# Oxidative Stress Promotes Asexual Reproduction and Apogamy in the Red Seaweed *Pyropia yezoensis*

**DOI:** 10.3389/fpls.2017.00062

**Published:** 2017-01-27

**Authors:** Megumu Takahashi, Koji Mikami

**Affiliations:** ^1^Department of Aquatic Bioscience, Faculty of Bio-Industry, Tokyo University of AgricultureAbashiri, Japan; ^2^Laboratory of Aquaculture, Genetics and Genomics, Division of Marine Life Science, Faculty of Fisheries Sciences, Hokkaido UniversityHakodate, Japan

**Keywords:** apogamy, oxidative stress, life cycle, generation switch, *Pyropia yezoensis*

## Abstract

The marine red seaweed *Pyropia yezoensis* has a haploid-diploid life cycle wherein two heteromorphic generations, a haploid gametophyte and a diploid sporophyte, are reciprocally generated from conchospores and carpospores, respectively. When we treated gametophytic blades of *P. yezoensis* with H_2_O_2_, discharge of asexual monospores was accelerated, resulting in increased numbers of gametophytic clones. Production of sporophytes without fertilization of male and female gametes was also observed. These findings indicate that oxidative stress can induce vegetative cells to develop into monospores that produce gametophytes asexually and can sometimes prompt carpospores to develop into sporophytes. The discovery of oxidative stress-triggered asexual reproduction and -apogamy will stimulate progress in studies of life-cycle regulation in *P. yezoensis*.

## Introduction

Plants are multicellular organisms that exhibit alternation of ontogenies, such as haploid gametophyte and diploid sporophyte generations, during their life cycles (Coelho et al., 2011a; Bowman et al., 2016; Horst and Reski, 2016), such that a single nuclear genome operates two different developmental programs (Friedman, 2013). Developmental programs for haploid and diploid generations are initiated by meiosis to produce haploid spores and fertilization of male and female gametes to produce diploid spores, respectively. However, homeotic mutations that induce apomixis, i.e., a switch between generation without fertilization or meiosis, have been reported in terrestrial plants (Bell, 1992; Schmidt et al., 2015). Apomixis encompasses two developmental processes, namely apospory (the occurrence of a gametophyte from a sporophyte without meiosis) and apogamy (the occurrence of a sporophyte from a gametophyte without fertilization). Thus, apomixis is a highly useful tool with which to elucidate the regulatory mechanisms of reprograming required for generation switching.

**FIGURE 1:**
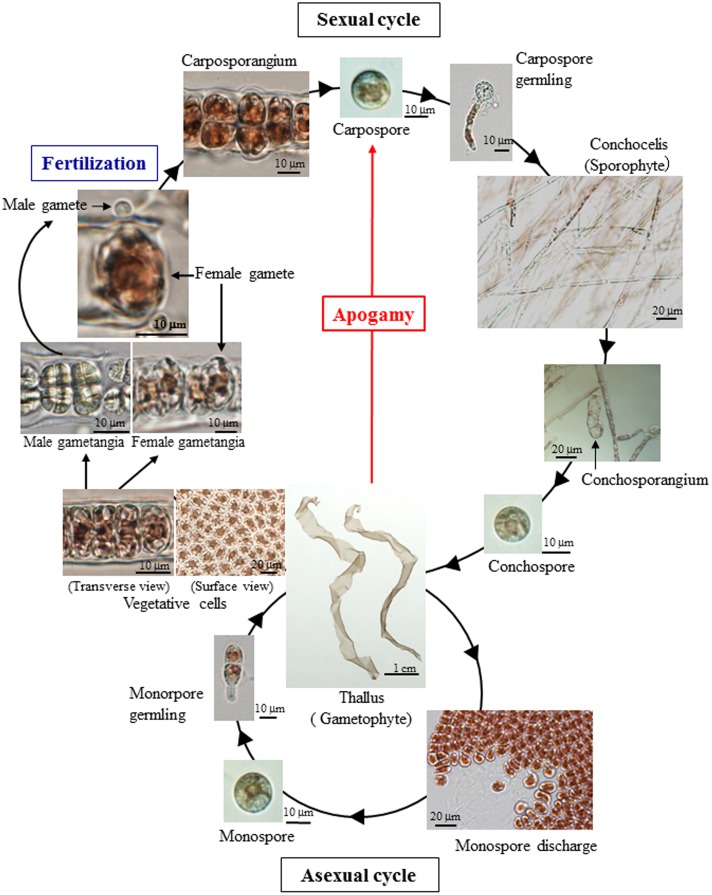
**Life cycle of the marine red seaweed *Pyropia yezoensis*.** Monospores and carpospores are released from an asexual gametophyte and fertilization-dependent carposporangium, respectively. However, apogamy produces carpospores directly from gametophyte without fertilization.

The life-cycle of the red seaweed *Pyropia yezoensis*, previously referred as *Porphyra yezoensis* and recently renamed according to the novel classification of Bangiales (Sutherland et al., 2011), has been extensively studied and comprises the reciprocal appearance of free-living haploid gametophytes and diploid sporophytes as leafy blades and filamentous conchocelis as shown in **Figure 1** (Sahoo et al., 2002; Shimizu et al., 2008; Blouin et al., 2011; Mikami et al., 2012; Herron et al., 2013). Like other organisms, *P. yezoensis* requires fertilization and meiosis for the transitions from gametophyte to sporophyte and from sporophyte to gametophyte, respectively (**Figure 1**). Despite the accumulation of knowledge about its life cycle, mechanisms regulating the generation-to-generation transitions in *P. yezoensis* have not been well studied to date. The present work sought to provide information about the effect of reactive oxygen species on *P. yezoensis* reproduction, which is not known. We here found that oxidative stress can promote the generation switch through apogamy in *P. yezoensis*.

## Materials and Methods

Gametophytic blades of *P. yezoensis* strain U-51 were cultured in PES medium, which was made using filtered natural seawater with PES [Provasoli’s enriched seawater; Provasoli (1968)] solution, under 60 μmol/m^2^/s irradiance with a photocycle of 10 h light and 14 h dark at 15°C. The PES medium was continuously bubbled with filter-sterilized air and renewed weekly. Gametophytes of ca. 10 mm length (whole blades) were used for experiments. H_2_O_2_ was dissolved in distilled water (DW) to create a 0.1 M stock solution. We employed total six blades per experiment by dividing into three sets (two individuals per set) to perform standing-culture using three upper wells of a 6-well culture dish (Iwaki Sci Tech Div., Asahi Techno Glass, Japan) containing 5 mL PES medium for 2 weeks at 15°C with addition of the H_2_O_2_ solution at working concentrations indicated in the text or DW corresponding to the maximum volume of the H_2_O_2_ stock solution. The concentration of the solutions did not exceed 1% after addition to the medium. Culture medium was renewed weekly by replacing gametophytes to a new well containing new medium. After H_2_O_2_ treatment, the numbers of monospore germlings, carpospore germlings and non-germinating spores in each well were counted under an inverted light microscope (CKX-41, Olympus, Tokyo, Japan) equipped with a camera (DP26, Olympus).

## Results and Discussion

**FIGURE 2:**
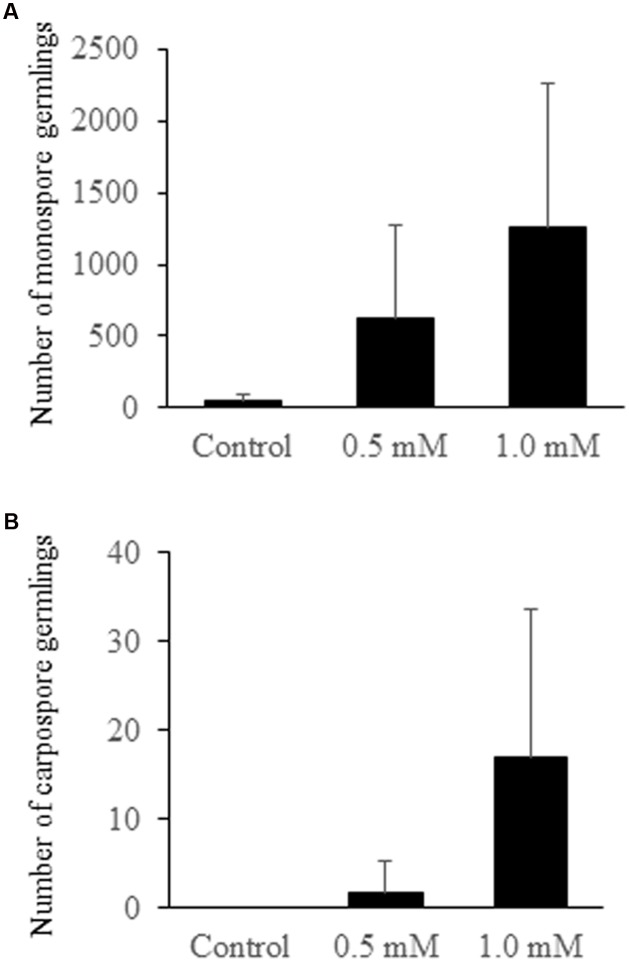
**Number of germlings from monospores and carpospores released from H_2_O_2_-treated gametophytic thallus.** **(A)** Monospore germlings. **(B)** Carpospore germlings. Error bars indicated ±SD of four independent experiments (see **Table 1**).

When each set of two *P. yezoensis* gametophytes was treated with 0, 0.5, or 1.0 mM H_2_O_2_, production and release of asexual monospores was accelerated (**Figure 2A**), although effects of H_2_O_2_ varied among experiments (**Table 1**). Thus, oxidative stress is one factor promoting monospore discharge for asexual propagation. In plants, it is well known that oxidative stress enhances photosynthesis (Foyer and Shigeoka, 2011) and stimulates Ca^2+^ influx (Mori and Schroeder, 2004; Rentel and Knight, 2004). We previously reported that a Ca^2+^ influx that requires photosynthetic activity is responsible for monospore discharge in *P. yezoensis* (Takahashi et al., 2010). Thus, it is possible that H_2_O_2_ treatment of gametophytic thallus activates photosynthesis-dependent Ca^2+^ influx to promote release of monospores. This possibility would suggest that *P. yezoensis* may harbor H_2_O_2_-dependent Ca^2+^ transporters.

**Table 1:** Number of germlings from monospores and carpospores released from H_2_O_2_-treated gametophytic thallus in four independent experiments.

Conditions	Monospore germlings	Carpospore germlings	Non-germinating spore
**Experiment 1**			
Control	91	0	5
0.5 mM H_2_O_2_	480	0	5
1.0 mM H_2_O_2_	1964	30	25
**Experiment 2**			
Control	1	0	0
0.5 mM H_2_O_2_	442	0	5
1.0 mM H_2_O_2_	687	2	25
**Experiment 3**			
Control	2	0	0
0.5 mM H_2_O_2_	37	0	0
1.0 mM H_2_O_2_	154	3	77
**Experiment 4**			
Control	92	0	6
0.5 mM H_2_O_2_	1555	7	17
1.0 mM H_2_O_2_	2240	33	20


*Two gametophytes were used for each assay (six individuals per experiment).*

**FIGURE 3:**
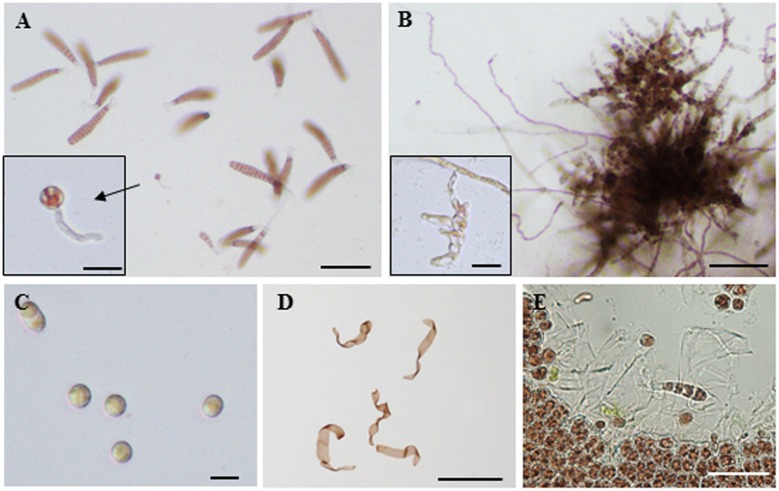
**Oxidative stress-triggered apogamy in *Pyropia yezoensis*.** **(A)** Sporophyte and gametophytes released from spores from H_2_O_2_-treated gametophytic thallus. Most spores were monospores that developed into gametophytes. Enlarged view shows a sporophyte. **(B)** Filamentous sporophyte and conchosporangia (enlarged view) developed from a carpospore produced by apogamy. **(C)** Conchospores produced from conchosporangia. **(D)** Gametophytes developed from conchospores. **(E)** Discharge of monospores from gametophytes developed from apogamy-derived conchospores and normally growing monospore germlings. Scale bars = 100 μm (**A,B,E**), 20 μm (**C** and enlarged views in **A** and **B**), and 1 mm **(D)**.

We also observed an induction of apogamy resulting in the production of sporophytes from released spores without development and fertilization of male and female gametes (**Figure 3A**), although at low-frequency (**Figure 2B**; **Table 1**). Since the apogamous sporophytes generated conchosporangia from which conchospores were produced and developed into normal gametophytes (**Figures 3B–E**), carpospores generated under oxidative stress conditions were indistinguishable from those produced in conchosporangia via the normal life cycle (**Figure 1**), suggesting diploidy of apogamous sporophytes, although it should be confirmed. Thus, oxidative stress has the potential to reprogram the developmental fate of a gametophytic vegetative cell to produce carpospores from which sporophytes develop. This finding represents the first evidence of abiotic stress-induced apogamy in seaweeds.

Previously, it was reported that sporophyte development from gametophytic cells could be artificially induced in the red seaweed *Pyropia pseudolinearis* when free cells were prepared by treatment of thallus with allantoin followed by homogenization (Saito et al., 2008). Allantoin is a purine metabolite (Bai et al., 2006) that activates jasmonic acid (JA) signaling in an abscisic acid (ABA)-dependent manner in *Arabidopsis thaliana* (Watanabe et al., 2014; Takagi et al., 2016). Although *P. yezoensis* lacks endogenous JA, it contains ABA (Mikami et al., 2016). It is possible that allantoin stimulates ABA biosynthesis in *P. yezoensis* cells, which might accelerate monospore production. In light of our results, wounding-dependent production of H_2_O_2_ might influence apogamy, because homogenization of allantoin-treated thallus was required for preparation of free cells (Saito et al., 2008). Indeed, sporophyte development by apogamy has been observed when protoplasts were prepared from gametophytes by artificial digestion of the cell wall (Waaland et al., 1990; Ar Gall et al., 1993). Our observation of H_2_O_2_-induced apogamy is consistent with these findings. Alternatively, the function of allantoin as a nitrogen source in algae (Anita et al., 1980; Prasad, 1983) suggests the involvement of nutritional conditions in apogamy in *P. yezoensis*. Therefore, it is necessary to examine whether ABA- and nitrogen-rich conditions induce apogamy in *P. yezoensis* and *P. pseudolinearis* to identify factors related to the initiation of apogamy in red seaweeds.

Elucidation of the relationship between the commitment to a developmental fate and the regulation of life-cycle progression is central to understanding the transitions between life-cycle generations. The H_2_O_2_-triggered apogamy identified in the present study suggests the presence of master regulators positively and negatively controlling ontogenies of life cycle generations in *P. yezoensis* as in terrestrial plants and the brown alga (Peters et al., 2008; Mosquna et al., 2009; Okano et al., 2009; Coelho et al., 2011b; Sakakibara et al., 2013; Horst et al., 2016). Since H_2_O_2_ treatment of released monospores did not produce any sporophytes (Takahashi and Mikami, unpublished), the fate of asexual spores appears to be fixed when they are released. Thus, we propose that precursors of thallus-derived unicellular spores have a competency to develop into both gametophyte and sporophyte, and that oxidative stress sometimes stimulates the selection of the conchospore developmental program before spore release. It is possible that distinct factors determining the early developmental process of monospores or carpospores before spore release might exist in *P. yezoensis*. In fact, we have already demonstrated that development of gametophytes starts with an asymmetrical cell division of the monospore to produce functionally distinct vegetative and rhizoid cells (Li et al., 2008) and also observed that filamentous conchocelis is produced by budding of the initial filament from a carpospore and subsequent elongation via symmetrical cell division and branching (unpublished). Therefore, reprogramming of developmental patterns might be closely related to switching of genetic programs regulating asymmetrical and symmetrical cell division. In addition, as in moss and brown seaweed (Peters et al., 2008; Mosquna et al., 2009; Okano et al., 2009; Coelho et al., 2011a,b; Sakakibara et al., 2013; Horst and Reski, 2016; Horst et al., 2016), it is possible that *P. yezoensis* has master regulators governing the expression of genes required for determination of gametophyte and sporophyte identities through asymmetrical and symmetrical cell division, respectively. However, these factors remain to be identified.

Identification of master regulators and their target genes, both of which would be involved in determination of the developmental fate of unicellular spores from thallus, in *P. yezoensis* would help in understanding the relationships between expression of genetic programs regulating ontogenies of each generation and activation of the master regulators. In this respect, the artificial induction of apogamy reported in the present study has the potential to provide a break-through model system. In fact, apogamy in red seaweeds has been reported in *Bangia fuscopurpurea* and *Pyropia haitanensis* as spontaneously occurring (Notoya and Iijima, 2003; Yan et al., 2007), suggesting that apogamy is a natural strategy for generation switching in certain red seaweeds. By contrast, *P. yezoensis* apparently lacks this strategy, which is an advantage for investigating the molecular mechanisms regulating transitions of life-cycle generations in non-apomictic plants, like *A. thaliana* and *Physcomitrella patens* (Mosquna et al., 2009; Okano et al., 2009; Sakakibara et al., 2013; Horst et al., 2016).

The oxidative stress-dependent apogamy we have discovered in *P. yezoensis* provides novel insight into the developmental plasticity of the transitions between gametophytes and sporophytes in the seaweed life cycle and could be a good model for the study of life-cycle regulation.

## Author Contributions

MT performed the experiments and collected the data. MT and KM designed the research, analyzed the data, and wrote the manuscript.

## Conflict of Interest Statement

The authors declare that the research was conducted in the absence of any commercial or financial relationships that could be construed as a potential conflict of interest.
